# dCCA: detecting differential covariation patterns between two types of high-throughput omics data

**DOI:** 10.1093/bib/bbae288

**Published:** 2024-06-18

**Authors:** Hwiyoung Lee, Tianzhou Ma, Hongjie Ke, Zhenyao Ye, Shuo Chen

**Affiliations:** Maryland Psychiatric Research Center, School of Medicine, University of Maryland, Baltimore, MD 21201, United States; The University of Maryland Institute for Health Computing (UM-IHC), North Bethesda, MD 20852, United States; Department of Epidemiology and Biostatistics, University of Maryland, College Park, MD 20742, United States; Department of Epidemiology and Biostatistics, University of Maryland, College Park, MD 20742, United States; The University of Maryland Institute for Health Computing (UM-IHC), North Bethesda, MD 20852, United States; Division of Biostatistics and Bioinformatics, Department of Epidemiology and Public Health, School of Medicine, University of Maryland, Baltimore, MD 21201, United States; Maryland Psychiatric Research Center, School of Medicine, University of Maryland, Baltimore, MD 21201, United States; The University of Maryland Institute for Health Computing (UM-IHC), North Bethesda, MD 20852, United States; Division of Biostatistics and Bioinformatics, Department of Epidemiology and Public Health, School of Medicine, University of Maryland, Baltimore, MD 21201, United States

**Keywords:** canonical correlation analysis, differential correlation, bipartite graph, multivariate-to-multivariate, multiomics, RNA gene regulation

## Abstract

**Motivation:**

The advent of multimodal omics data has provided an unprecedented opportunity to systematically investigate underlying biological mechanisms from distinct yet complementary angles. However, the joint analysis of multi-omics data remains challenging because it requires modeling interactions between multiple sets of high-throughput variables. Furthermore, these interaction patterns may vary across different clinical groups, reflecting disease-related biological processes.

**Results:**

We propose a novel approach called Differential Canonical Correlation Analysis (dCCA) to capture differential covariation patterns between two multivariate vectors across clinical groups. Unlike classical Canonical Correlation Analysis, which maximizes the correlation between two multivariate vectors, dCCA aims to maximally recover differentially expressed multivariate-to-multivariate covariation patterns between groups. We have developed computational algorithms and a toolkit to sparsely select paired subsets of variables from two sets of multivariate variables while maximizing the differential covariation. Extensive simulation analyses demonstrate the superior performance of dCCA in selecting variables of interest and recovering differential correlations. We applied dCCA to the Pan-Kidney cohort from the Cancer Genome Atlas Program database and identified differentially expressed covariations between noncoding RNAs and gene expressions.

**Availability and Implementation:**

The R package that implements dCCA is available at https://github.com/hwiyoungstat/dCCA.

## Introduction

Multiomics data have recently gained increased attention due to their multifaceted involvement in various aspects of the underlying biological environment. For example, in cancer research, the joint analysis of gene expression and non-coding RNAs (ncRNAs) that are not translated into proteins, including microRNAs (miRNAs), long noncoding RNAs (lncRNAs), and circular RNAs (circRNAs), has become a promising avenue to uncover the pivotal functional role of ncRNAs in cancer. ncRNA may display both tumor suppressive and oncogenic functions, and aberrant expression of ncRNAs can induce abnormal transcriptional regulation in critical tumor-related genes, which ultimately contribute to tumor initiation and progression. Existing studies focused on a few specific ncRNAs and their regulatory roles in a small set of genes without fully utilizing the information from the multiomics data generated by high-throughput technology [1, 2]. Gaining a comprehensive picture of the association between non-coding RNAs and genes at a transcriptome-wide level is imperative to advance our knowledge of cancer pathogenesis. In practical applications, the combined analysis of two types of omics data (such as gene expression and microbiome, or metabolomics and microbiome, among various combinations) offers a novel approach to comprehending the intricacies and interactive nature of biological systems. Despite the potentially valuable findings from multiomics data, the joint analysis of two sets of high-dimensional variables raises computational challenges.

Canonical Correlation Analysis (CCA), originally introduced by [3], is widely used to assess associations between two sets of multivariate data [4, 5]. As common covariation among variables may exist within each set of multivariate data, CCA aims to identify latent factors for both multivariate vectors that maximize the correlations between them. As a popular model to decipher the interactions between two sets of multivariate data, CCA has been widely applied to a wide range of biomedical data analysis [6]. The resulting canonical variables (i.e. factors) by CCA facilitate visualization and effectively reveal associations between two distinct data blocks in a lower dimensional space. Furthermore, they can serve as input features in various tasks, including classification, particularly in situations where the use of the original variables is challenging due to multicollinearity and high dimensionality [7].

Conventionally, CCA is only applicable to multivariate vectors with a dimensionality lower than the sample size due to the singularity of the sample covariance matrices [8]. The recent advances in statistical methods, e.g. various versions of sparse CCA methods (sCCA) [9, 10] have been developed to alleviate this dimensionality constraint by utilizing regularization techniques that ensure algorithmic stability and promote parsimony for enhanced interpretability. However, challenges remain for sCCA methods to identify the underlying differential multivariate-to-multivariate association patterns across clinical groups. For example, the associations between ncRNAs and gene expressions can exhibit variations influenced by factors such as different cancer stages, and subtypes, thereby introducing significant heterogeneity. Neither classic CCA nor sCCA methods can capture the underlying differential covariation patterns [11], which motivates our current research.

To address this unmet need, we propose a new differential Canonical Correlation Analysis (dCCA) method to identify the heterogeneity in multivariate-to-multivariate associations across groups with different clinical or experimental conditions. We propose a novel objective function that maximizes the multivariate-to-multivariate correlations while recognizing inter-group discrepancy. By relaxing multiple constraints imposed on the covariance matrices, we implement the objective function using a subgradient-based algorithm. Additionally, to address the high dimensionality of both data blocks, we introduce a bipartite dense graph-based screening procedure.

The rest of this paper is organized as follows. In Method, we introduce the details of dCCA method and conduct extensive simulation studies to assess its performance by comparing it with competing methods. In Results, we apply the method to data obtained from the Cancer Genome Atlas Program (TCGA) database to explore the association between noncoding RNA and gene expression in kidney cancer. The paper concludes in Conclusion with a discussion.

## Method

In this study, we consider a multivariate-multivariate dataset comprising $n$ observations. The dataset consists of two high-dimensional data blocks of dimensions $p$ and $q$, respectively, denoted by $\mathbf{X} \in \mathbb{R}^{n\times p}$ and $\mathbf{Y}\in \mathbb{R}^{n \times q}$. We first consider the case where $\max (p,q)<n$, and for the case where $\max (p,q)\geq n$, we resort to a screening procedure (see Screening) to reduce dimensionality. Additionally, a binary group variable $\mathbf{Z}$, which takes values of either 0 or 1, serves as a moderator, differentiating the association patterns between $\mathbf{X}$ and $\mathbf{Y}$. Based on $\mathbf{Z}$, we divide the complete data $(\mathbf{X}, \mathbf{Y}) \in \mathbb{R}^{n\times (p+q)}$ into two subsets: $(\mathbf{X}_{0}, \mathbf{Y}_{0})\in \mathbb{R}^{n_{0} \times (p+q)}$ and $(\mathbf{X}_{1}, \mathbf{Y}_{1})\in \mathbb{R}^{n_{1} \times (p+q)}$, where the subscripts indicate the corresponding $\mathbf{Z}$ values. Here, $n_{0}$ and $n_{1}$ represent the numbers of participants in groups $\mathbf{Z}=0$ and $\mathbf{Z}=1$, respectively. For example, in our data application, $\mathbf{X}$ represents microRNA (miRNA) data, $\mathbf{Y}$ represents gene expression data, and $\mathbf{Z}$ represents distinct subtypes of kidney cancer, where $\mathbf{Z}=0$ corresponds to a common subtype and $\mathbf{Z}=1$ corresponds to a rare subtype.

### dCCA (Association analysis)

Our primary objective is two-fold: (i) to assess whether underlying association patterns exist between two sets of high-dimensional variables $\mathbf{X}$ and $\mathbf{Y}$ by maximally revealing the common patterns and (ii) further to identify differential associations between $\mathbf{X}$ and $\mathbf{Y}$ for those with $\mathbf{Z}=0$ vs. $\mathbf{Z}=1$. To achieve the first objective, we can employ the classic CCA with the objective function represented as follows: 


\begin{align*} \mathop{\arg\max}\limits_{\mathbf{u} \in \mathbb{R}^{p},\mathbf{v} \in \mathbb{R}^{q}} \text{Cor}(\mathbf{X}\mathbf{u}, \mathbf{Y}\mathbf{v}), \end{align*}

where $\mathbf{u}\in \mathbb{R}^{p}$ and $\mathbf{v}\in \mathbb{R}^{q}$ are loading vectors that assign the weights to the original variables in the datasets $\mathbf{X}$ and $\mathbf{Y}$, respectively.

To address the second objective, we consider the differences in the canonical correlations between two subgroups categorized by the value of $\mathbf{Z}$, represented as 


\begin{align*} \mathop{\arg\max}\limits_{\mathbf{u} \in \mathbb{R}^{p},\mathbf{v} \in \mathbb{R}^{q}} \vert \text{Cor}(\mathbf{X}_{0}\mathbf{u}, \mathbf{Y}_{0}\mathbf{v}) - \text{Cor}(\mathbf{X}_{1}\mathbf{u}, \mathbf{Y}_{1}\mathbf{v}) \vert. \end{align*}

This maximizes the discrepancy in association patterns across distinct subsets, allowing us to gain insights into the heterogeneity of the association patterns between subgroups. Therefore, to simultaneously achieve both goals, we propose the dCCA approach with an integrated objective function


\begin{align*} \mathop{\arg\max}\limits_{\mathbf{u} \in \mathbb{R}^{p},\mathbf{v} \in \mathbb{R}^{q}} \text{Cor}(\mathbf{X}\mathbf{u}, \mathbf{Y}\mathbf{v}) + \lambda \left(\vert \text{Cor}(\mathbf{X}_{0}\mathbf{u}, \mathbf{Y}_{0}\mathbf{v}) - \text{Cor}(\mathbf{X}_{1}\mathbf{u}, \mathbf{Y}_{1}\mathbf{v}\right) \vert). \end{align*}

We can rewrite the objective function as 


(1) \begin{align*} \begin{aligned} &\mathop{\arg\max}\limits_{\mathbf{u} \in \mathbb{R}^{p},\mathbf{v} \in \mathbb{R}^{q}} \frac{\mathbf{u}^{\top} \boldsymbol{\Sigma}_{\mathbf{X}\mathbf{Y}}\mathbf{v}}{\sqrt{\mathbf{u}^{\top} \boldsymbol{\Sigma}_{\mathbf{X}}\mathbf{u}}\sqrt{\mathbf{v}^{\top} \boldsymbol{\Sigma}_{\mathbf{Y}}\mathbf{v}}}\\ &+ \lambda \left\vert \frac{\mathbf{u}^{\top} \boldsymbol{\Sigma}_{\mathbf{X}_{0}\mathbf{Y}_{0}}\mathbf{v}}{\sqrt{\mathbf{u}^{\top} \boldsymbol{\Sigma}_{\mathbf{X}_{0}}\mathbf{u}}\sqrt{\mathbf{v}^{\top} \boldsymbol{\Sigma}_{\mathbf{Y}_{0}}\mathbf{v}}} - \frac{\mathbf{u}^{\top} \boldsymbol{\Sigma}_{\mathbf{X}_{1}\mathbf{Y}_{1}}\mathbf{v}}{\sqrt{\mathbf{u}^{\top} \boldsymbol{\Sigma}_{\mathbf{X}_{1}}\mathbf{u}}\sqrt{\mathbf{v}^{\top} \boldsymbol{\Sigma}_{\mathbf{Y}_{1}}\mathbf{v}}} \right\vert, \end{aligned}\end{align*}

where $p \times q$ matrices $\boldsymbol{\Sigma }_{\mathbf{X}\mathbf{Y}}$, $\boldsymbol{\Sigma }_{\mathbf{X}_{0}\mathbf{Y}_{0}}$, and $\boldsymbol{\Sigma }_{\mathbf{X}_{1}\mathbf{Y}_{1}}$ represent the cross-covariance matrices of the respective pairs of data: $(\mathbf{X}, \mathbf{Y})$, $(\mathbf{X}_{0}, \mathbf{Y}_{0})$, and $(\mathbf{X}_{1}, \mathbf{Y}_{1})$. Additionally, $\boldsymbol{\Sigma }_{\mathbf{X}}$, $\boldsymbol{\Sigma }_{\mathbf{X}_{0}}$, $\boldsymbol{\Sigma }_{\mathbf{X}_{1}}$, $\boldsymbol{\Sigma }_{\mathbf{Y}}$, $\boldsymbol{\Sigma }_{\mathbf{Y}_{0}}$, and $\boldsymbol{\Sigma }_{\mathbf{Y}_{1}}$ denote the covariance matrices of each individual dataset ($\mathbf{X}$, $\mathbf{X}_{0}$, $\mathbf{X}_{1}$, $\mathbf{Y}$, $\mathbf{Y}_{0}$, and $\mathbf{Y}_{1}$).

In summary, both CCA and dCCA aim to identify vectors $\mathbf{u} \in \mathbb{R}^{p}, \mathbf{v} \in \mathbb{R}^{q}$. The goal of CCA is to maximize the correlation between $\mathbf{X}\mathbf{u}$ and $\mathbf{Y}\mathbf{v}$ for all groups. In contrast, while maintaining this primary objective, dCCA also aims to maximize the difference in correlations between two groups ($\mathbf{Z}=0$ vs $\mathbf{Z}=1$), i.e., $\text{Cor}(\mathbf{X}_{0}\mathbf{u}, \mathbf{Y}_{0}\mathbf{v})$ vs $\text{Cor}(\mathbf{X}_{1}\mathbf{u}, \mathbf{Y}_{1}\mathbf{v})$.

**Figure 1:**
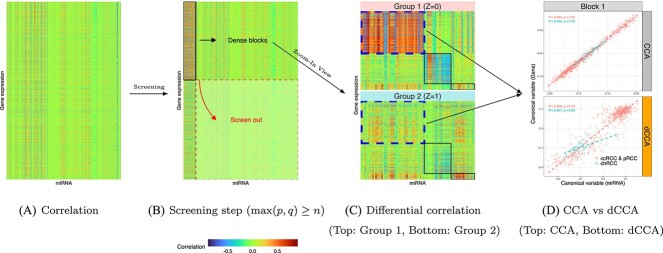
The demonstration of dCCA workflow: (**A**) is the heatmap of marginal correlation matrix between two vectors of high-dimensional variables $\mathbf{X}$ and $\mathbf{Y}$; each row represents a gene expression variable, and each column represents a miRNA variable; (**B**) shows the heatmap in (**A**) after a screening step when the dimensionality of $\mathbf{X}$ and $\mathbf{Y}$ is high; non-informative variables $\mathbf{X}$ and $\mathbf{Y}$ can be excluded for further analysis; (**C**) the differential correlation patterns between the two clinical groups are demonstrated in the enlarged heatmaps; we next perform the dCCA analysis on postscreened $\mathbf{X}$ and $\mathbf{Y}$ to compute $\mathbf{u}$ and $\mathbf{v}$, and (**D**) illustrates the contrasting results of the canonical variables from $\mathbf{X}$ and $\mathbf{Y}$ in dCCA vs CCA within the first block. Specifically, dCCA can better identify differential correlation patterns between the two clinical groups; note that the screening step in (**B**) is not necessary when $\max (p,q)<n$.

The objective function (1) can simultaneously identify the underlying correlation patterns for both groups and highlight the differential correlations between groups. These two terms are linked by a tuning parameter $\lambda>0$. Thus, $\lambda $ plays a crucial role in balancing the classical CCA term and the discrepancy term. Specifically, a higher value of $\lambda $ places a stronger emphasis on the between-group discrepancy, whereas a smaller $\lambda $ leads to results more similar to the classic CCA. We adopt the commonly used cross-validation strategy to objectively select the optimal $\lambda $ [12]. The canonical variables (i.e. $\mathbf{X}\mathbf{u} \in \mathbb{R}^{n}$ and $\mathbf{Y}\mathbf{v} \in \mathbb{R}^{n}$) in (1) are used to reduce the dimensions for both multivariate vectors and highlight the latent correlation patterns (see Fig. 1C).

***Implementation.*** We numerically optimize the objective function as follows. The numerators involve the cross-covariance between $\mathbf{X}$ and $\mathbf{Y}$, capturing the correlation between these two sets of variables and forming a primary focus of our analysis. The denominators, which encompass the normalization of $\mathbf{u}$ and $\mathbf{v}$ using their respective covariance matrices, ensure that their contributions are scaled relative to the variability (or covariance) of the data.

By reformulating the above using the constraint form commonly employed in CCA, it can be expressed as


\begin{align*} \mathop{\arg\max}\limits_{\mathbf{u} \in \mathbb{R}^{p},\mathbf{v} \in \mathbb{R}^{q}} && &\mathbf{u}^{\top} \boldsymbol{\Sigma}_{\mathbf{X}\mathbf{Y}}\mathbf{v} + \lambda \left(\left\vert \mathbf{u}^{\top} \boldsymbol{\Sigma}_{\mathbf{X}_{0}\mathbf{Y}_{0}}\mathbf{v} - \mathbf{u}^{\top} \boldsymbol{\Sigma}_{\mathbf{X}_{1}\mathbf{Y}_{1}}\mathbf{v} \right\vert \right)\\ \hbox{such that} \ && &\mathbf{u}^{\top} \boldsymbol{\Sigma}_{\mathbf{X}}\mathbf{u} =\mathbf{u}^{\top} \boldsymbol{\Sigma}_{\mathbf{X}_{0}}\mathbf{u} = \mathbf{u}^{\top} \boldsymbol{\Sigma}_{\mathbf{X}_{1}}\mathbf{u} = 1,\\ && &\mathbf{v}^{\top} \boldsymbol{\Sigma}_{\mathbf{Y}}\mathbf{v} =\mathbf{v}^{\top} \boldsymbol{\Sigma}_{\mathbf{Y}_{0}}\mathbf{v} = \mathbf{v}^{\top} \boldsymbol{\Sigma}_{\mathbf{Y}_{1}}\mathbf{v} = 1. \end{align*}

The above objective function retains its focus on maximizing the correlation between linear combinations while incorporating the regularization term that accounts for the difference between two groups. All constraints from the denominators in the original objective function in (1) aim to ensure the loading vectors $\mathbf{u}$ and $\mathbf{v}$ possessing unit length within distinct covariance structures associated with subsets of data ($\mathbf{X}, \mathbf{X}_{0}, \mathbf{X}_{1}, \mathbf{Y},\mathbf{Y}_{0}$, and $\mathbf{Y}_{1}$). However, optimizing the above while simultaneously satisfying all the constraints is computationally intractable. Following a commonly used numerical optimization strategy by [9] and [13], we relax the constraints by substituting all covariance matrices within the constraints with identity matrices of the same dimensions. Consequently, the modified objective function becomes


(2) \begin{align*} \begin{aligned} &\mathop{\arg\max}\limits_{\mathbf{u} \in \mathbb{R}^{p},\mathbf{v} \in \mathbb{R}^{q}} \mathbf{u}^{\top} \boldsymbol{\Sigma}_{\mathbf{X}\mathbf{Y}} \mathbf{v} + \lambda \vert\mathbf{u}^{\top} \left(\boldsymbol{\Sigma}_{\mathbf{X}_{0}\mathbf{Y}_{0}} - \boldsymbol{\Sigma}_{\mathbf{X}_{1}\mathbf{Y}_{1}}\right) \mathbf{v}\vert\\ &\hbox{such that} \ \Vert\mathbf{u}\Vert^{2} = 1, \Vert\mathbf{v}\Vert^{2} = 1. \end{aligned}\end{align*}

By reformulating the constraint optimization problem in (2) using the Lagrangian function (i.e. $\mathcal{L}(\mathbf{u},\mathbf{v},\alpha ,\beta ) = \mathbf{u}^{\top }\boldsymbol{\Sigma }_{\mathbf{X}\mathbf{Y}}\mathbf{v} + \lambda \vert \mathbf{u}^{\top } (\boldsymbol{\Sigma }_{\mathbf{X}_{0}\mathbf{Y}_{0}}-\boldsymbol{\Sigma }_{\mathbf{X}_{1}\mathbf{Y}_{1}})\mathbf{v}\vert + \frac{\alpha }{2}(\mathbf{u}^{\top }\mathbf{u}-1) + \frac{\beta }{2}(\mathbf{v}^{\top }\mathbf{v}-1) $), we develop an optimization algorithm in Algorithm 1.


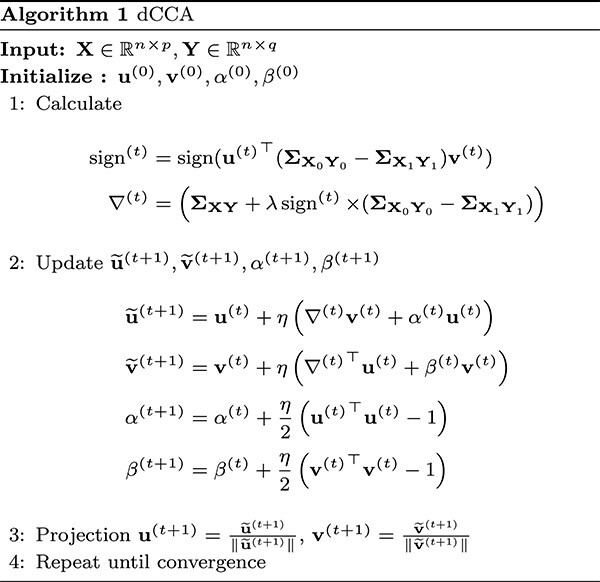


### Screening

When the dimensionality of $\mathbf{X}$ and $\mathbf{Y}$ is higher than the sample size $n$ or non-informative noise presents, we can first conduct a screening step to exclude inactive pairs before implementing the objective function (2). This alleviates computational limitations for CCA and facilitates a more efficient study by narrowing down the focus to a subset of variables of interest. Following [14], many screening methods have been developed across diverse contexts, each tailored to accomplish its specific objectives. For example, [15] developed a screening procedure for two high-dimensional variables. In this research, we introduce a novel graph-based screening process to efficiently identify active pairs of variables between high-dimensional $\mathbf{X}$ and high-dimensional $\mathbf{Y}$.

We present the association between $\mathbf{X}$ and $\mathbf{Y}$ as a bipartite graph, denoted as $G = (\mathcal{N}_{x}, \mathcal{N}_{y}, \mathcal{E})$, where $\mathcal{N}_{x}$ and $\mathcal{N}_{y}$ represent distinct node sets for $\mathbf{X}$ and $\mathbf{Y}$, respectively (i.e. $\vert \mathcal{N}_{x} \vert =p, \vert \mathcal{N}_{y} \vert =q$, where $\vert \cdot \vert $ denote the cardinality of the set), and $\mathcal{E} $ denotes the edges (i.e. $\mathcal{E} = \{e_{xy} \in \mathcal{E} \vert x\in \mathcal{N}_{x}, y \in \mathcal{N}_{y}\}$). Assuming that associations between $\mathbf{X}$ and $\mathbf{Y}$ are concentrated in highly correlated pairs of nodes rather than occurring across the entire set of pairs, we extract $k$ quasi-bicliques which are subsets of pairs of nodes with dense associations and filter out the irrelevant variables (i.e. screening).

Let $A \in \mathbb{R}^{p \times q}$ be the biadjacency matrix with entries $A_{ij} = I(\vert W_{ij} \vert> r)$, obtained by thresholding the weighted edge matrix of the bipartite graph (e.g. the absolute $\mathbf{X}-\mathbf{Y}$ correlation matrix: $W_{ij}= \vert \text{Cor}(\mathbf{X}_{.i},\mathbf{Y}_{.j})\vert $) with the threshold value $r$. Then, the $k$th quasi-biclique consisting of the node set $(\mathcal{N}_{x}^{k}, \mathcal{N}_{y}^{k})$ is obtained by optimizing the following objective function: 


(3) \begin{align*} \mathop{\arg\max}\limits_{\substack{ \mathcal{N}_{x}^{k} \subset \mathcal{N}_{x} \setminus \bigcup_{j=0}^{k-1} \mathcal{N}_{x}^{j}\\ \mathcal{N}_{y}^{k} \subset \mathcal{N}_{y} \setminus \bigcup_{j=0}^{k-1} \mathcal{N}_{y}^{j}}} \frac{\Vert A_{k} \Vert_{1,1}}{(\vert \mathcal{N}_{x}^{k} \vert \vert \mathcal{N}_{y}^{k} \vert)^{\lambda_{k}}},\end{align*}

where $\mathcal{N}_{x}^{0}=\mathcal{N}_{y}^{0}= \emptyset $. Note that $A_{k}\in \mathbb{R}^{\vert \mathcal{N}_{x}^{k}\vert \times \vert \mathcal{N}_{y}^{k} \vert }$ is the biadjacency matrix of the subgraph induced by the nodes ($\mathcal{N}_{x}^{k}$, $\mathcal{N}_{y}^{k}$), and $\Vert \cdot \Vert _{1,1}$ is the entry-wise $L_{1,1}$ norm (i.e. $\Vert A_{k} \Vert _{1,1} = \sum _{j \in \mathcal{N}_{y}^{k}}\sum _{i \in \mathcal{N}_{x}^{k}} \vert A_{ij}\vert $). To implement the above screening procedure, we utilize a greedy algorithm [16], and the algorithm’s summary is provided in Algorithm 2 (see details for the algorithm in the Supplementary Material).


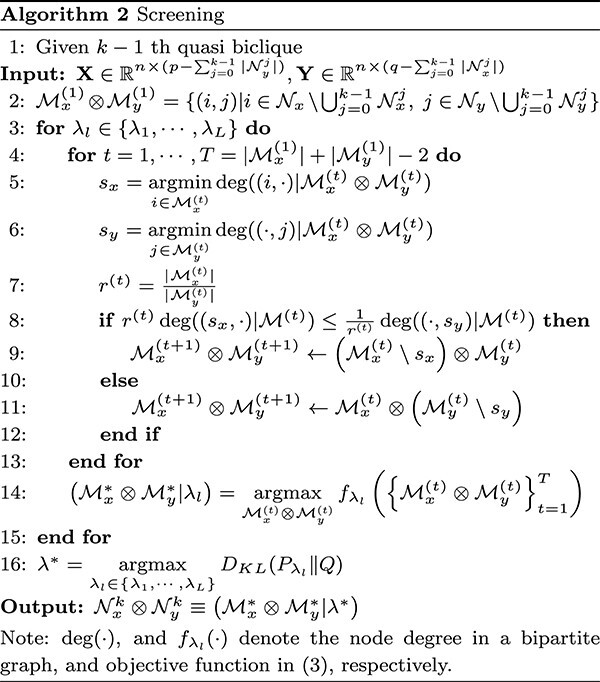


The tuning parameter $\lambda _{k}$ in (3) plays a crucial role in extracting the dense subset $\mathcal{N}_{x}^{k} \otimes \mathcal{N}_{y}^{k}$. For example, large $\lambda _{k}$ tends to yield a more parsimonious result, characterized by reduced size and increased density of $\mathcal{N}_{x}^{k} \otimes \mathcal{N}_{y}^{k}$. We select the optimal $\lambda _{k}$ in a data-driven manner using the Kullback–Leibler (KL) divergence. Specifically, considering two distinct blocks; (i) the dense block ($\mathcal{N}_{x}^{k}, \mathcal{N}_{y}^{k}$), and (ii) outside the dense block ($\mathcal{N}_{x} \setminus \bigcup _{j=0}^{k} \mathcal{N}_{x}^{j}, \mathcal{N}_{y} \setminus \bigcup _{j=0}^{k} \mathcal{N}_{y}^{j}$), where within the dense block, $A_{ij}$ is more likely to be 1 for a pair of variables $i$ and $j$ with a strong association, while outside the dense block, it is likely to be 0 (i.e. uncorrelated). Therefore, the binarized association strength indicator variable $A_{ij}$ can be assumed to follow a mixture of Bernoulli distribution, i.e. $P(A_{ij})=\delta _{ij} \hbox{\texttt{Bernoulli}} (\pi _{1}) + (1-\delta _{ij}) \hbox{\texttt{Bernoulli}} (\pi _{0})$, where $\delta _{ij}=I((i,j)\in \mathcal{N}_{x}^{k} \otimes \mathcal{N}_{y}^{k})$. Alternatively, one can consider a reference Bernoulli distribution $Q(A_{ij})=\hbox{\texttt{Bernoulli}}(\pi )$, assuming that $\mathbf{X}-\mathbf{Y}$ pairs exhibit no clustered patterns, where the dense bipartite graph-based screening is not effective. As the KL divergence quantifies the dissimilarity between the well-modeled distribution $P$ (representing the dense pattern), and the naive distribution $Q$ (i.e. $D_{KL}(P_{\lambda }\Vert Q) = \sum _{i,j} P_{\lambda }(A_{ij})\log \frac{P_{\lambda }(A_{ij})}{Q(A_{ij})}$), it serves as a suitable measure for selecting the tuning parameter $\lambda $. Thus, we select the tuning parameter $\lambda $ by maximizing the following KL divergence: 


(4) \begin{multline*} \lambda_{k} = \mathop{\arg\max}\limits_{\lambda} D_{KL}(P_{\lambda} \Vert Q)\\ \sum_{(i,j)\in \mathcal{N}_{x}^{k} \otimes \mathcal{N}_{y}^{k}} \left( A_{ij}\pi_{1} \log \frac{{\pi}_{1}}{{\pi}} + (1-A_{ij})(1-{\pi}_{1}) \log \frac{(1-{\pi}_{1})}{(1-{\pi})} \right)\\ + \sum_{(i,j)\notin \in \mathcal{N}_{x}^{k} \otimes \mathcal{N}_{y}^{k}} \left( A_{ij}{\pi}_{0} \log \frac{{\pi}_{0}}{{\pi}} + (1-A_{ij})(1-{\pi}_{0}) \log \frac{(1-{\pi}_{0})}{(1-{\pi})} \right). \ \end{multline*}

The Bernoulli distribution parameters (i.e. $\pi , \pi _{1}, \pi _{0}$) can be estimated using maximum likelihood estimation; see the Supplementary Material for details.

By filtering out non-informative signals (potential noise or weak associations), the remaining quasi-bicliques can better reveal the latent (differential) correlation patterns. Therefore, the screening step can generally reduce the computational cost and improve the estimation accuracy. However, when the dimensions of variables $\mathbf{X}$ and $\mathbf{Y}$ are moderate (e.g. less than the sample size, i.e. $\max (p,q) <n$) and the noise level is low, dCCA can be performed without the screening step.

### Simulation

In this section, we conduct simulation studies to evaluate the performance of dCCA with the screening procedure (dCCA_+Screen_) and benchmark it with comparable multivariate analysis methods, including sparse CCA (SCCA), sparse PCA (SPCA), and sparse LDA (SLDA, [17] implemented in the sparseLDA package). Both SCCA and SPCA are based on the unified penalized matrix decomposition framework in [9] and are implemented through the PMA package in R. In addition, to assess the effectiveness of the screening procedure, we also use the unscreened version of dCCA as a competing method. For CCA and SPCA, we performed stratified analyses by applying the methods separately to each group. SCCA_Sep_ and SPCA_Sep_ denote these separate applications, respectively. Within these methods, subscripts 0 and 1 indicate the groups corresponding to $\mathbf{Z}=0$ and $\mathbf{Z}=1$, respectively.

We simulate the multivariate predictors $\mathbf{X}$ from a $p$-dimensional multivariate normal distribution (i.e. $\mathtt{Normal}({\textbf{0}},\boldsymbol{\Sigma }_{p})$). By introducing a binary group label $\mathbf{Z}=\{0,1\}$, which serves as a moderator in the association between $\mathbf{X}$ and $\mathbf{Y}$, we generate the multivariate response $\mathbf{Y}$ (gene expression) from two different $q$-dimensional multivariate normal distributions: $\mathtt{Normal}(\mathbf{X} \mathbf{B}_{0},\boldsymbol{\Sigma }_{q} \vert \mathbf{Z}=0)$ and $\mathtt{Normal}(\mathbf{X} \mathbf{B}_{1},\boldsymbol{\Sigma }_{q}\vert \mathbf{Z}=1)$. Here, $\mathbf{B}_{0}$ and $\mathbf{B}_{1}$ represent the $p \times q$ regression coefficients matrices corresponding to subgroups where $\mathbf{Z}$ equals $0$ or $1$, respectively. Additionally, we use equal group sizes with $n_{0}=n_{1}=200$.

We set the dimensions to $p = 200$ and $q = 400$. Among all possible pairs of $p \times q$ associations, we specify the active pairs within two dense blocks: the first block is sized $p_{1}=10$ and $q_{1}=20$, and the second block is sized $p_{2}=5$ and $q_{2}=10$. Non-zero values are assigned to the entries within these dense blocks of the coefficient matrix, while the remaining entries are set to zero. This configuration is designed to replicate the circuitry commonly observed in RNA gene regulation networks, wherein the RNA-gene pairs within the dense block exhibit concentrated interactions, while the inactive pairs outside the block do not play a role in influencing gene expression through RNA.

Under this multi-dense block configuration, we consider two settings. In the first, the direction (sign) of the association differs depending on $\mathbf{Z}$, while in the second, the association between $\mathbf{X}$ and $\mathbf{Y}$ only exists when $\mathbf{Z}=1$. In both settings, the non-zero coefficients within $\mathbf{B}_{1}$ are assigned negative values, while the first scenario involves positive coefficients in $\mathbf{B}_{0}$, and coefficients of 0 in $\mathbf{B}_{0}$ for the second scenario. Our simulation settings are designed to emulate biologically plausible scenarios. In the first scenario, different clinical statuses can lead to contrasting regulatory effects of RNA on gene expression. The second scenario reflects the selective and condition-dependent nature of association in biological systems, where the association between RNA and gene expression is present only under specific clinical statuses.

We assess the performance for all methods by two criteria: (i) variable selection accuracy and (ii) recovery of the differential correlation patterns between groups. Specifically, we evaluate the accuracy of $p_{0}$ and $q_{0}$ selection using precision, recall, and $F_{1}$ score. To assess how precisely the method recovers differential correlation patterns, we calculate the absolute bias (i.e. $\vert \rho _{j}-\widehat{\rho }_{j}\vert $) for different groups separately. Here, $\widehat{\rho }_{j} = \text{\text{Cor}}(\mathbf{X}_{j}\widehat{\mathbf{u}}, \mathbf{Y}_{j}\widehat{\mathbf{v}})$ represents the estimated canonical correlation of the $j$th subgroup, while $\rho _{j}$ represents the theoretical correlation under the noiseless case. Thus, in the first and second settings, we have $\rho _{0}$ equal to 1 and 0, respectively, while $\rho _{1}=-1$ remains fixed in both scenarios. Moreover, we assess whether canonical variables derived from dCCA and comparable methods can distinguish the two groups. We fitted a logistic regression with the group as the outcome and canonical variables as predictors. Performance was evaluated by comparing the Areas Under the Curve (AUC) of the Receiver Operating Characteristic (ROC) curves.

**Figure 2:**
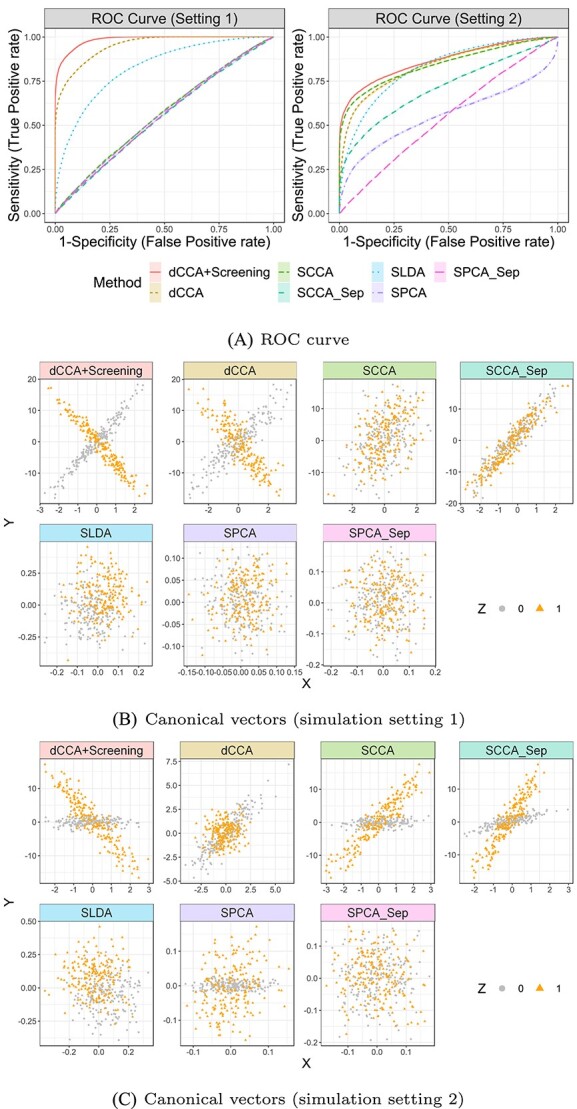
Results of simulation studies: (**A**) ROC curves compare the performance of the methods’ canonical variables in the classification task; the middle and bottom panels display scatter plots of the projected (canonical) variables from different methods for (**B**) Setting 1 and (**C**) Setting 2, respectively.

Results are summarized in Tables 1 and 2 and displayed in Fig. 2, with 100 replications per simulation setting. Regarding variable selection, dCCA_+Screen_ accurately identifies non-zero, correlated variables in both data blocks $\mathbf{X}$ and $\mathbf{Y}$ for both settings (see $F_{1}$ scores), and outperforms the competing methods. SCCA exhibits a high rate of false positives across all settings, resulting in low precision. SPCA accurately selects variables in $\mathbf{Y}$ but not in $\mathbf{X}$. SLDA misses most true variables because it is designed for classification instead of variable selection.

**Table 1:** Simulation Results (Setting 1: The direction of the association pattern is opposite between groups): We compare dCCA with the screening procedure (dCCA_+Screen_) to dCCA without the screening procedure (dCCA), and three competing methods (sparse CCA (SCCA), sparse LDA (SLDA), and sparse PCA (SPCA)); SCCA_Sep_ and SPCA_Sep_ are used to denote these separate applications, respectively; subscripts 0 and 1 denote the groups corresponding to $\mathbf{Z}=0$, and $\mathbf{Z}=1$, respectively.

Variable selection in $\mathbf{X}$ ($p_{0}$ selection)			
Method	Precision	Recall	$\boldsymbol{F_{1}}$
dCCA_+Screen_	**0.8582 (0.14)**	**0.9920 (0.03)**	**0.9121 (0.11)**
SCCA	0.0809 (0.04)	0.1700 (0.09)	0.1094 (0.06)
SCCA_Sep_0__	0.2645 (0.03)	0.7173 (0.05)	0.3856 (0.04)
SCCA_Sep_1__	0.2590 (0.03)	0.7207 (0.06)	0.3803 (0.04)
SLDA	0.0607 (0.06)	0.0607 (0.06)	0.0607 (0.06)
SPCA	0.0760 (0.05)	0.1560 (0.10)	0.1019 (0.06)
SPCA_Sep_0__	0.0771 (0.05)	0.1573 (0.10)	0.1031 (0.06)
SPCA_Sep_1__	0.0692 (0.04)	0.1407 (0.09)	0.0925 (0.06)
Variable selection in $\mathbf{Y}$ ($q_{0}$ selection)			
Method	Precision	Recall	$F_{1}$
dCCA_+Screen_	0.9878 (0.09)	**1.0000 (0.00)**	**0.9899 (0.09)**
SCCA	0.2159 (0.04)	0.7547 (0.09)	0.3352 (0.05)
SCCA_Sep_0__	0.1548 (0.02)	0.8140 (0.09)	0.2600 (0.03)
SCCA_Sep_1__	0.1556 (0.02)	0.8063 (0.10)	0.2607 (0.04)
SLDA	0.1093 (0.05)	0.1093 (0.05)	0.1093 (0.05)
SPCA	**1.0000 (0.00)**	0.6543 (0.02)	0.7909 (0.01)
SPCA_Sep_0__	**1.0000 (0.00)**	0.6517 (0.02)	0.7889 (0.01)
SPCA_Sep_1__	**1.0000 (0.00)**	0.6507 (0.02)	0.7882 (0.01)
Identifying correlation and classification ($\rho _{0}=1, \rho _{1}=-1$)			
Method	$\vert \rho _{0}-\widehat{\rho }_{0}\vert $	$\vert \rho _{1}-\widehat{\rho }_{1}\vert $	AUC
dCCA_+Screen_	**0.0342 (0.02)**	**0.0352 (0.02)**	**0.9831 (0.01)**
dCCA	0.0938 (0.01)	0.1138 (0.02)	0.9498 (0.01)
SCCA	0.5722 (0.11)	1.4408 (0.1)	0.5601 (0.04)
SCCA_Sep_	0.0629 (0.01)	1.9353 (0.02)	0.5433 (0.03)
SLDA	1.0018 (0.07)	0.9951 (0.07)	0.8241 (0.02)
SPCA	1.0043 (0.12)	1.0113 (0.12)	0.5585 (0.04)
SPCA_Sep_	1.0300 (0.12)	0.9847 (0.12)	0.5486 (0.03)

**Table 2:** Simulation Results (Setting 2: The association between $\mathbf{X}$ and $\mathbf{Y}$ exists only in one clinical group, specifically when $\mathbf{Z}=1$): We compare dCCA with the screening procedure (dCCA_+Screen_) to dCCA without the screening procedure (dCCA), and three competing methods (sparse CCA (SCCA), sparse LDA (SLDA), and sparse PCA (SPCA)); SCCA_Sep_ and SPCA_Sep_ are used to denote these separate applications, respectively; subscripts 0 and 1 denote the groups corresponding to $\mathbf{Z}=0$, and $\mathbf{Z}=1$, respectively.

Variable selection in $\mathbf{X}$ ($p_{0}$ selection)			
Method	Precision	Recall	$\boldsymbol{F_{1}}$
dCCA_+Screen_	**0.8620 (0.09)**	**0.9680 (0.05)**	**0.9083 (0.06)**
SCCA	0.2564 (0.04)	0.7207 (0.06)	0.3772 (0.04)
SCCA_Sep_0__	0.0752 (0.04)	0.1627 (0.09)	0.1026 (0.05)
SCCA_Sep_1__	0.2585 (0.03)	0.7193 (0.06)	0.3796 (0.04)
SLDA	0.0627 (0.06)	0.0627 (0.06)	0.0627 (0.06)
SPCA	0.0788 (0.05)	0.1620 (0.10)	0.1057 (0.07)
SPCA_Sep_0__	0.0766 (0.05)	0.1567 (0.10)	0.1025 (0.06)
SPCA_Sep_1__	0.0690 (0.04)	0.1407 (0.09)	0.0924 (0.06)
Variable selection in $\mathbf{Y}$ ($q_{0}$ selection)			
Method	Precision	Recall	$F_{1}$
dCCA_+Screen_	0.9994 (0.00)	**0.9930 (0.02)**	**0.9961 (0.01)**
SCCA	0.1593 (0.02)	0.8103 (0.09)	0.2661 (0.03)
SCCA_Sep_0__	0.0731 (0.03)	0.1587 (0.07)	0.0999 (0.04)
SCCA_Sep_1__	0.1559 (0.02)	0.8067 (0.10)	0.2612 (0.04)
SLDA	0.1063 (0.05)	0.1063 (0.05)	0.1063 (0.05)
SPCA	**1.0000 (0.00)**	0.6520 (0.02)	0.7892 (0.01)
SPCA_Sep_0__	0.0765 (0.05)	0.0837 (0.05)	0.0795 (0.05)
SPCA_Sep_1__	**1.0000 (0.00)**	0.6507 (0.02)	0.7882 (0.01)
Identifying correlation and classification ($\rho _{0}=0, \rho _{1}=-1$)			
Method	$\vert \rho _{0}-\widehat{\rho }_{0}\vert $	$\vert \rho _{1}-\widehat{\rho }_{1}\vert $	AUC
dCCA_+Screen_	0.1030 (0.06)	**0.0754 (0.03)**	**0.8648 (0.02)**
dCCA	0.5532 (0.34)	1.2009 (0.69)	0.8369 (0.05)
SCCA	0.1798 (0.07)	1.9327 (0.02)	0.8423 (0.02)
SCCA_Sep_	0.8091 (0.02)	1.9354 (0.02)	0.7116 (0.02)
SLDA	0.0583 (0.04)	0.9958 (0.07)	0.8237 (0.01)
SPCA	**0.0562 (0.04)**	1.0188 (0.12)	0.5580 (0.04)
SPCA_Sep_	0.0631 (0.05)	0.9889 (0.12)	0.5465 (0.02)

When capturing differential correlation patterns between groups, dCCA_+Screen_ generally demonstrates the least absolute bias, except when $\mathbf{Z}=0$ in setting 2, where $\rho _{0}=0$. In this setting, SPCA achieves the best performance; however, dCCA shows a nearly comparable performance. Note that the satisfactory performance of SLDA and SPCA in this specific case stems from their inherent design, which does not prioritize uncovering association patterns between two multivariate data blocks. Consequently, they consistently yield near-zero correlations in all settings, which leads to a significant bias in every other case. This renders their projected variables lacking meaningful interpretation (see Fig. 2). Due to the absence of addressing group heterogeneity, conventional SCCA produced nearly identical canonical correlations for both groups in setting 1. Furthermore, SCCA cannot distinguish the direction (sign) of the overall association for different groups and generated positively correlated canonical variables when $\mathbf{Z}=1$ (see (B) in Fig. 2), even though the true underlying correlation is negative. This results in a significant bias (see $\vert \rho _{1}-\widehat \rho _{1}\vert $ in Table 1 and Table 2). Performing SCCA and SPCA separately (i.e. SCCA_Sep_ and SPCA_Sep_) for each clinical group misses the underlying differential association patterns between groups, resulting in high correlation estimation biases. In contrast, dCCA_+Screen_ accurately discerns the underlying correlation between the two groups.

In addition, the canonical variables obtained from dCCA_+Screen_ achieve the highest AUC in both settings, demonstrating their advantage in the classification task over those derived from other dimension-reduction techniques.

In summary, dCCA method outperforms the benchmark multivariate association analysis models in accurately selecting active pairs of variables and identifying distinct underlying association patterns for different groups. The dCCA-derived canonical variables can also classify groups with improved accuracy.

***Assessing robustness of dCCA*:** We further examine whether dCCA introduces false positive differential correlations when the cross-group differential association pattern is absent. In this setting, we simulate identical regression coefficient matrices, i.e. $\mathbf{B}_{0}=\mathbf{B}_{1}$ within the same multi-block structure employed in the previous simulation settings, and assess false positive findings.

The results in Table 3 demonstrate that the false positive rate (FPR) is below 5% for dCCA_+Screen_. The results for correlation estimation and variable selection are provided in Table S1 of the Supplementary Material.

**Table 3:** FPR; we test the difference in canonical vectors between groups under the test level $\alpha =0.05$.

Method	dCCA_+Screen_	dCCA	SCCA	SCCA_Sep_
FPR	0.04	0.16	0.07	0.37
Method	SLDA	SPCA	SPCA_Sep_	
FPR	0.04	0.02	0.23	

## Results

We applied our method to Pan-kidney cohort data obtained from TCGA. This cohort offers a wide array of datasets, including gene expression, non-coding RNA (e.g. long noncoding RNAs (lncRNAs) and microRNAs (miRNAs)), along with clinical information (e.g. cancer stage and subtypes), enabling comprehensive research into kidney cancer. In our analysis, we uncover how the association between miRNAs and gene expression is influenced by different cancer subtypes. RNA sequencing was used for miRNA data (in RPM) and gene expression data (in RPKM), both of which were downloaded from LinkedOmics [18]. We conducted data preprocessing steps. Specifically, for the miRNA data, we excluded miRNAs with zero expression across all samples and applied $\log 2$ transformation to stabilize variance and make the data more symmetrically distributed. In gene expression data, genes with low expression levels are regarded as uninformative. Therefore, we applied a mean expression cutoff of $5$ to filter out such genes, enabling us to prioritize those with robust expression levels. The processed dataset contains a total of $n=680$ observations and has dimensions of $p=719$ and $q=1340$ for miRNAs and genes, respectively.

Renal cell carcinoma (RCC) is the predominant form of kidney cancer in adults and is categorized into various subtypes based on histopathological characteristics. In samples from the Pan-Kidney cohort, three subtypes are identified: Clear cell renal cell carcinoma (ccRCC), Papillary renal cell carcinoma (pRCC), and Chromophobe renal cell carcinoma (chRCC). Each of these subtypes exhibits unique cancer progression patterns, genetic traits, and RNA profiles, which, in turn, can impact gene and RNA regulation differently. The first two subtypes (ccRCC and pRCC) are common types, collectively accounting for 85%–95% of RCC cases, while chRCC is a rare subtype that accounts for $\sim $ 5% of all RCC cases. In our analysis, we treat these kidney cancer types as the group variable, assigning $\mathbf{Z}=0$ to common kidney cancer types (ccRCC and pRCC), and $\mathbf{Z}=1$ for chRCC, a rare kidney cancer type. The number of subjects in each cancer subtype is $n_{0}=614$ for the common subtype and $n_{1}=66$ for the rare subtype.

**Figure 3:**
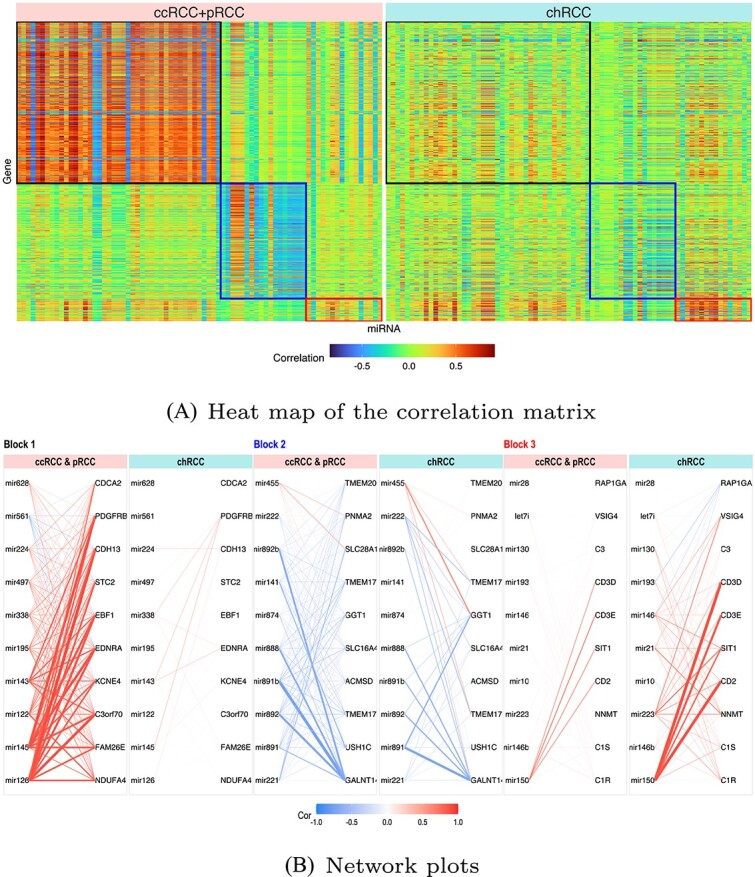
(**A**) Heat map illustrating the difference in the correlation matrix (miRNAs vs. genes) between different subtypes (common vs. rare) within the dense blocks; (**B**) network plots: in each block, nodes to the left represent the top 10 miRNAs, while nodes to the right represent the top 10 genes; the top 10 miRNAs and genes were chosen based on the summation of the absolute values of elements within each column (miRNA) and row (gene) of the corresponding block in the correlation matrix; the direction (sign) of the association is denoted by different colors (positive: red, negative: blue) in the edges, while the strength of the connection is visualized through both the width and transparency of the edge.

**Figure 4:**
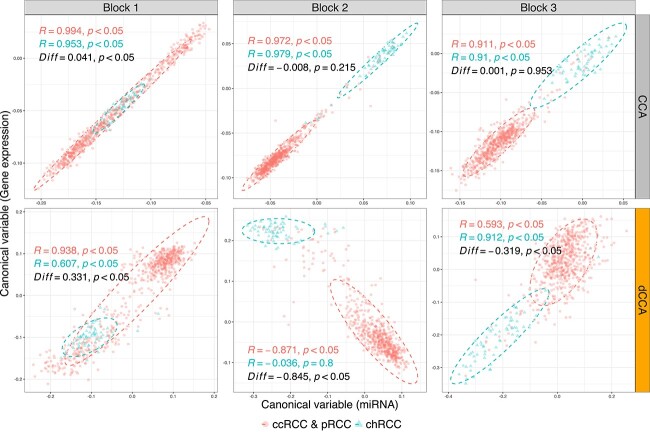
Comparison of scatter plots of canonical variables: miRNA (on the $x$-axis) vs. gene expression (on the $y$-axis) obtained from CCA in the upper panel and dCCA in the lower panels (orange strip); different subtypes of kidney cancer are visually distinguished by color (red for common subtypes (ccRCC and pRCC), and blue for chRCC); Pearson correlation coefficients (*R*) separately calculated from each subtype and their corresponding $p$-values (*p*) are given and color-coded similarly as above; in addition, the statistical significance of the difference (*Diff*) in canonical correlations between the two subtypes is tested, and the associated $p$-values are given (in black).

Since both $p$ and $q>n$ in this study, we first perform the screening step of dCCA. The screening procedure filters out non-informative pairs of miRNAs and genes and retains 77 miRNA variables and 591 gene variables. These variables comprises three ($k=3$) bipartite blocks (see Fig. 3): Block 1: 43 miRNAs $\times $ 319 genes; Block 2: 18 miRNAs $\times $ 227 genes; and Block 3: 16 miRNAs $\times $ 45 genes. In each block, distinct differential association patterns are present between the two cancer subtypes (see Fig. 3). In Block 1 (upper left corner), miRNA and gene are stronger (positively) correlated in the common cancer type group than in the chRCC group. Block 2 also demonstrates stronger (negatively) correlations for the common cancer type group in comparison to the chRCC group. Contrastingly, in Block 3, the correlations are stronger for the chRCC group compared with the common subtype. We then implement the objective function of dCCA on the filtered data to assess the differentially expressed $\mathbf{X}-\mathbf{Y}$ correlation patterns between groups. The produced canonical variables (see Fig. 4) reflect the differential correlation patterns in three blocks. In Block 1, both groups exhibit positive correlations between canonical variables, with a stronger strength observed in the common subtypes (ccRCC, pRCC) in comparison to chRCC. In Block 2, canonical correlation is negative for the common subtypes, whereas it is close to zero for chRCC. In Block 3, the correlation associated with chRCC is stronger than that of the common subtypes.

These findings are well aligned with results from prior studies. For example, block 1 identifies miRNA-gene pairs that are tightly connected in ccRCC and pRCC subtypes but loosely connected in the chRCC subtype. We searched two existing databases, miRCancer [19  http://mircancer.ecu.edu] and dbDEMC [20  https://www.biosino.org/dbDEMC/index]. Most miRNAs in Block 1 (e.g. miR-126, miR-145, miR-122) were identified as critical and differentially expressed in the ccRCC subtype but not in the chRCC subtype (see the Supplementary Material). In Block 2, miR-141, a unique miRNA signature in clear cell RCC [21], was found to be associated with critical tumor suppressor genes such as *USH1C* [22] in common RCC subtypes, while it was not associated in rare RCC subtypes. We also performed pathway analysis on the identified genes and found that several genes in Block 3 (e.g. *CD3D, CD3E, CD2, SIT1*) were enriched in pathways related to T cell and lymphocyte activation (see the Supplementary Material). dCCA identified miR-150, a miRNA that plays critical roles in lymphocyte development and is significantly associated with RCC survival [23, 24]. It was found to be strongly co-expressed with these genes in the chRCC subtype but weakly in the ccRCC and pRCC subtypes. A stronger miR-150-immune gene regulatory bond in chRCC may explain its utility in prognosis of RCC survival compared with the ccRCC and pRCC subtypes [25]. In addition, miR-223 in Block 3, a cancer–specific survival-related biomarker [26, 27], was found to be associated with genes in chRCC but not in other kidney cancer subtypes.

In comparison, we also apply the classic CCA[4] and sCCA[9] to this dataset. However, neither of the two methods identifies the underlying differential correlation patterns extracted by dCCA. For example, in Fig. 4, the correlations between canonical variables by CCA are almost identical between the two clinical groups, which misses the group differences potentially reflecting differential biological mechanisms.

## Conclusion

We have developed a new multivariate-to-multivariate analysis tool, dCCA, to decipher the complex interaction patterns between two types of high-dimensional omics data. We focus on extracting differentially expressed omics-to-omics interaction patterns between clinical groups. dCCA, unlike classic CCA and SCCA methods, more effectively uncovers interaction patterns between two types of omics data that are related to clinical status. Thus, the differential interaction patterns identified by dCCA can help pinpoint potential biomarkers that distinguish the subtypes. This approach may also provide insights into the distinct biological mechanisms that differ between groups. For example, identifying subtype-specific mechanisms may suggest targeted therapeutic strategies for the disease. However, our study remains exploratory rather than confirmatory, future studies and experiments need to be performed to further validate the findings. For example, an *in vitro* approach by growing cell lines from each cancer type may further validate our findings.

dCCA is computationally efficient and can handle the interactions between thousands-to-thousands variables as the graph-based screening procedure can efficiently filter non-informative features. For validation, we applied dCCA to an additional dataset (breast cancer study in TCGA). See the Supplementary Material for details.

The proposed dCCA method currently focuses on analyzing datasets with binary group variables. Expanding dCCA to accommodate group variables with more than two categories, such as the four molecular subtypes of breast cancer (HER2-enriched, Luminal A, Luminal B, Basal-like), will significantly enhance its utility for analyzing more complex datasets. For example, applying this method to datasets involving patients at different stages of cancer can provide insights into uncovering ordinal trends and dynamically varying association patterns throughout the progression of the disease. We provide a potential two-step solution for handling more than two groups in the Supplementary Material.

**Key Points**

- dCCA deciphers the complex interaction patterns between two types of high-dimensional omics data.
- Specifically dCCA extracts differentially expressed omics-to-omics interaction patterns between clinical groups, which can provide insights into the distinct biological mechanisms that differ between groups.
- We propose a novel graph-based approach to efficiently identify active variable pairs in two high-dimensional spaces (i.e. $\mathbf{X}$ and $\mathbf{Y}$), outperforming existing methods in accurately selecting variables within both $\mathbf{X}$ and $\mathbf{Y}$.

## Supplementary Material

supp_material_dCCA_bbae288

## Data Availability

The miRNA and gene expression data utilized in this study are accessible through the Cancer Genome Atlas Program (TCGA) Pan-kidney cohort via the website https://www.cancer.gov/ccg/research/genome-sequencing/tcga.

## Availability and Implementation

The software package that implements dCCA is available at https://github.com/hwiyoungstat/dCCA.

## Author Biographies

**Hwiyoung Lee** is a postdoctoral research fellow at the Department of Epidemiology and Public Health, School of Medicine, University of Maryland, Baltimore.

**Tianzhou Ma** is an assistant professor at the Department of Epidemiology and Biostatistics, School of Public Health, University of Maryland, College Park.

**Hongjie Ke** is a PhD candidate at the Department of Epidemiology and Biostatistics, School of Public Health, University of Maryland, College Park.

**Zhenyao Ye** is a PhD candidate at the Department of Epidemiology and Public Health, School of Medicine, University of Maryland, Baltimore.

**Shuo Chen** is a professor at the Department of Epidemiology and Public Health, School of Medicine, University of Maryland, Baltimore.
