# Supplementary material for: dCCA: detecting differential covariation patterns between two types of high-throughput omics data
Source: Brief Bioinform. 2024 Jun 18;25(4):bbae288. doi: 10.1093/bib/bbae288 (PMC11184902; doi:10.1093/bib/bbae288)
Supplement: supp_material_dCCA_bbae288 [file supp_material_dcca_bbae288.pdf]

Brifiengs in Bioinformatics, , 1–7
doi: Supplementary Material

Supplementary Material: dCCA: detecting differential covariation patterns between two types of high-throughput omics data

Hwiyoung Lee,[1] Tianzhou Ma,[2] Hongjie Ke,[2] Zhenyao Ye[3] and Shuo Chen[1,3]*

[1]Maryland Psychiatric Research Center, School of Medicine, University of Maryland, Baltimore, MD 21201, USA, [2]Department of Epidemiology and Biostatistics, University of Maryland, College Park, MD 20742, USA and [3]Department of Epidemiology and Public Health, University of Maryland, School of Medicine, MD 21201, USA

Abstract

This document presents the supplementary information for our research titled "dCCA: Detecting Differential Covariation Patterns Between two types of high-throughput omics data

Details for Algorithm 2

In this section, we provide further details of Algorithm 2.

Definitions

$\deg(\cdot)$ denotes the degree function. Specifically, we define $\deg(i, \cdot) = \sum_{j=1}^{q} A_{ij}$, $\deg(\cdot, j) = \sum_{i=1}^{p} A_{ij}$, where A biadjacency matrix with p rows and q columns and an entry A_{ij} measures the correlation strength between the i-th variable in $\mathbf{X}$ and the j-th variable in $\mathbf{Y}$ ($i = 1, \cdots, p$ and $j = 1, \cdots, q$). Each variable $\mathbf{X}_i$ or $\mathbf{Y}_j$ can be considered as a node in a bipartite graph and A_{ij} is the edge connecting between them. $\deg(i, \cdot)$ can be calculated from the row sum of the i-th row in A, which represents the sum of correlations (edges) between $\mathbf{X}_i$ and all variables of $\mathbf{Y}$. Similarly, $\deg(\cdot, j)$ refers to the column sum of all correlations (edges) between $\mathbf{Y}_j$ and all variables of $\mathbf{X}$.

$f_{\lambda_l} = \frac{\|A_k\|_{1,1}}{(|\mathcal{N}_x^k||\mathcal{N}_y^k|)^{\lambda_l}}$ is the fraction which objective function (3) aims to maximize for the screening procedure. f_{λ_l} represents the criteria to extract the dense blocks. λ_l is a tuning parameter in the search space of $\lambda_l, \cdots, \lambda_L$ in algorithm 2. λ_k in (3) denotes the optimal λ_l by search in Algorithm 2. A_k denotes the k-th block biadjacency that Algorithm 2 aims to extract ($\|\cdot\|_{1,1}$ is the entry-wise $L_{1,1}$ norm), $|\mathcal{N}_x^k|$, and $|\mathcal{N}_y^k|$ represent the cardinalities of node sets of $\mathbf{X}$, and $\mathbf{Y}$ for the k-th dense block, respectively. f_{λ_l} can be computed based on each extracted block using Algorithm 2 (Tsourakakis et al. (2013); Wu et al. (2022)).

Estimation

The Bernoulli parameters in (4) can be estimated by using the maximum likelihood estimation. Specifically, π is estimated based on the entire bipartite graph, while π_1, π_0 are estimated based on the edges within and outside the dense block, respectively. For $i \in \mathcal{N}_x \setminus \bigcup_{j=0}^{k-1}$, and $j \in \mathcal{N}_y \setminus \bigcup_{j=0}^{k-1}$, the parameters in Bernoulli distributions can be estimated by

$$\pi_1 = \sum_{i,j} \frac{\delta_{ij} A_{ij}}{|\mathcal{N}_x^k||\mathcal{N}_y^k|},$$

$$\pi_0 = \sum_{i,j} \frac{(1-\delta_{ij}) A_{ij}}{(|\mathcal{N}_x \setminus \bigcup_{j=0}^{k-1} \mathcal{N}_x^j||\mathcal{N}_y \setminus \bigcup_{j=0}^{k-1} \mathcal{N}_y^j|) - (|\mathcal{N}_x^k||\mathcal{N}_y^k|)},$$

$$\pi = \sum_{i,j} \frac{A_{ij}}{(|\mathcal{N}_x \setminus \bigcup_{j=0}^{k-1} \mathcal{N}_x^j||\mathcal{N}_y \setminus \bigcup_{j=0}^{k-1} \mathcal{N}_y^j|)}.$$

© The Author . Published by Oxford University Press. All rights reserved. For permissions, please e-mail: journals.permissions@oup.com

Two-step dCCA Approach for Multiple Subgroups ($K > 2$)

In this section, we present a potential solution for applying dCCA to general cases involving $K > 2$ groups.

Step 1: Pair-wise comparisons

Perform pair-wise comparisons by applying dCCA.

$$\underset{\mathbf{u}\in\mathbb{R}^p,\mathbf{v}\in\mathbb{R}^q}{\operatorname{argmax}} \operatorname{Cor}(\mathbf{Xu},\mathbf{Yv}) + \lambda|\operatorname{Cor}(\mathbf{X}_k\mathbf{u},\mathbf{Y}_k\mathbf{v}) - \operatorname{Cor}(\mathbf{X}_l\mathbf{u},\mathbf{Y}_l\mathbf{v})| \text{ for } 1 \leq k < l \leq K$$

Because the differential interaction patterns between the two types of omics data may involve different subsets of biological measures (i.e., $\mathbf{u}$ and $\mathbf{v}$ can be different across pairs). For the breast cancer example, the biological mechanisms (interactions between the two omics datasets) in the Basal-like subtype may differ from those in the Luminal A, Luminal B, and HER2-enriched subtypes. Therefore, conducting pairwise comparisons as the initial step appears to be a valid approach.

Step 2: dCCA for general K subgroups

If differences are observed, then we perform dCCA to investigate further across all K subgroups. Here, we propose the dCCA for general $K > 2$ subgroups:

$$\underset{\mathbf{u}\in\mathbb{R}^p,\mathbf{v}\in\mathbb{R}^q}{\operatorname{argmax}} \operatorname{Cor}(\mathbf{Xu},\mathbf{Yv}) + \lambda \sum_{1\leq k<l\leq K} |\operatorname{Cor}(\mathbf{X}_k\mathbf{u},\mathbf{Y}_k\mathbf{v}) - \operatorname{Cor}(\mathbf{X}_l\mathbf{u},\mathbf{Y}_l\mathbf{v})| \tag{1}$$

The above objective function in (1) can be optimized by the similar optimization technique summarized in the main manuscript. In Algorithm S1, we provide the early version of the implementation strategy for optimizing (1).

Algorithm S1 dCCA for multi-subgroup

Input: $\mathbf{X} \in \mathbb{R}^{n\times p}, \mathbf{Y} \in \mathbb{R}^{n\times q}$
Initialize : $\mathbf{u}^{(0)}, \mathbf{v}^{(0)}, \alpha^{(0)}, \beta^{(0)}$
1: Calculate

$$\operatorname{sign}^{(t)}_{(k,l)} = \operatorname{sign}\left(\mathbf{u}^{(t)\top}\left(\mathbf{\Sigma}_{\mathbf{X}_k\mathbf{Y}_k} - \mathbf{\Sigma}_{\mathbf{X}_l\mathbf{Y}_l}\right)\mathbf{v}^{(t)}\right)$$
$$\nabla^{(t)} = \left(\mathbf{\Sigma}_{\mathbf{XY}} + \lambda\left(\sum_{1\leq k<l\leq K} \operatorname{sign}^{(t)}_{(k,l)}(\mathbf{\Sigma}_{\mathbf{X}_k\mathbf{Y}_k} - \mathbf{\Sigma}_{\mathbf{X}_l\mathbf{Y}_l})\right)\right)$$

2: Update $\widetilde{\mathbf{u}}^{(t+1)}, \widetilde{\mathbf{v}}^{(t+1)}, \alpha^{(t+1)}, \beta^{(t+1)}$

$$\widetilde{\mathbf{u}}^{(t+1)} = \mathbf{u}^{(t)} + \eta\left(\nabla^{(t)}\mathbf{v}^{(t)} + \alpha^{(t)}\mathbf{u}^{(t)}\right)$$
$$\widetilde{\mathbf{v}}^{(t+1)} = \mathbf{v}^{(t)} + \eta\left(\nabla^{(t)\top}\mathbf{u}^{(t)} + \beta^{(t)}\mathbf{v}^{(t)}\right)$$
$$\alpha^{(t+1)} = \alpha^{(t)} + \frac{\eta}{2}\left(\mathbf{u}^{(t)\top}\mathbf{u}^{(t)} - 1\right)$$
$$\beta^{(t+1)} = \beta^{(t)} + \frac{\eta}{2}\left(\mathbf{v}^{(t)\top}\mathbf{v}^{(t)} - 1\right)$$

3: Projection $\mathbf{u}^{(t+1)} = \frac{\widetilde{\mathbf{u}}^{(t+1)}}{\|\widetilde{\mathbf{u}}^{(t+1)}\|}$, $\mathbf{v}^{(t+1)} = \frac{\widetilde{\mathbf{v}}^{(t+1)}}{\|\widetilde{\mathbf{v}}^{(t+1)}\|}$
4: Repeat until convergence

Additional Simulation results

In this section, we provide the additional results of the simulation studies. Specifically, the results of simulation setting 3 are summarized in Table S1.

Table S1. Simulation Results (Setting 3: There is no differential pattern in the association between $\mathbf{X}$ and $\mathbf{Y}$ across the groups.): We compare dCCA with the screening procedure ($\text{dCCA}_{+\text{Screen}}$) to dCCA without the screening procedure (dCCA), and three competing methods (sparse CCA (SCCA), sparse LDA (SLDA), and sparse PCA (SPCA)). SCCA_{Sep} and SPCA_{Sep} are used to denote these separate applications, respectively. Subscripts 0 and 1 denote the groups corresponding to $\mathbf{Z} = 0$, and $\mathbf{Z} = 1$, respectively.

Variable selection in $\mathbf{X}$ (p_0 selection)			
Method	Precision	Recall	F_1
$\text{dCCA}_{+\text{Screen}}$	**0.9925 (0.02)**	**0.9907 (0.03)**	**0.9913 (0.02)**
SCCA	0.2191 (0.03)	0.7487 (0.06)	0.3384 (0.04)
$\text{SCCA}_{\text{Sep}_0}$	0.2645 (0.03)	0.7173 (0.05)	0.3856 (0.04)
$\text{SCCA}_{\text{Sep}_1}$	0.2596 (0.03)	0.7193 (0.05)	0.3808 (0.04)
SLDA	0.0607 (0.06)	0.0607 (0.06)	0.0607 (0.06)
SPCA	0.0760 (0.05)	0.1560 (0.10)	0.1019 (0.06)
$\text{SPCA}_{\text{Sep}_0}$	0.0771 (0.05)	0.1573 (0.10)	0.1031 (0.06)
$\text{SPCA}_{\text{Sep}_1}$	0.0692 (0.04)	0.1407 (0.09)	0.0925 (0.06)
Variable selection in $\mathbf{Y}$ (q_0 selection)			
Method	Precision	Recall	F_1
$\text{dCCA}_{+\text{Screen}}$	**1.0000 (0.00)**	**1.0000 (0.00)**	**1.0000 (0.00)**
SCCA	0.1252 (0.01)	0.8623 (0.08)	0.2186 (0.02)
$\text{SCCA}_{\text{Sep}_0}$	0.1548 (0.02)	0.8140 (0.09)	0.2600 (0.03)
$\text{SCCA}_{\text{Sep}_1}$	0.1561 (0.02)	0.8113 (0.09)	0.2617 (0.04)
SLDA	0.1157 (0.05)	0.1157 (0.05)	0.1157 (0.05)
SPCA	**1.0000 (0.00)**	0.6503 (0.02)	0.7879 (0.01)
$\text{SPCA}_{\text{Sep}_0}$	**1.0000 (0.00)**	0.6517 (0.02)	0.7889 (0.01)
$\text{SPCA}_{\text{Sep}_1}$	**1.0000 (0.00)**	0.6497 (0.02)	0.7874 (0.02)
Identifying correlation and difference ($\rho_0 = 1, \rho_1 = 1$)			
Method	$\lvert\rho_0 - \widehat{\rho}_0\rvert$	$\lvert\rho_1 - \widehat{\rho}_1\rvert$	$\lvert\widehat{\rho}_0 - \widehat{\rho}_1\rvert$
$\text{dCCA}_{+\text{Screen}}$	**0.0317 (0.02)**	**0.0309 (0.02)**	**0.0050 (0.01)**
dCCA	0.1025 (0.02)	0.1017 (0.02)	0.0243 (0.03)
SCCA	0.0427 (0.01)	0.0429 (0.01)	0.0070 (0.01)
SCCA_{Sep}	0.0629 (0.01)	0.0640 (0.02)	0.0175 (0.02)
SLDA	0.9982 (0.07)	1.0058 (0.08)	0.0729 (0.09)
SPCA	1.0047 (0.12)	1.0109 (0.12)	0.0818 (0.1)
SPCA_{Sep}	1.0300 (0.12)	1.0148 (0.12)	0.1457 (0.18)

Application of dCCA to TCGA BRCA data

In this section, we applied the dCCA method to The Cancer Genome Atlas Breast Invasive Carcinoma (TCGA-BRCA) data (`https://portal.gdc.cancer.gov/projects/TCGA-BRCA`). Our analysis focused on two specific subtypes: we compared the Basal-like subtype with a combined group of Luminal A and Luminal B subtypes. We have a total of $n = 744$ subjects, with $n_0 = 603$ belonging to the Luminal A & B subtypes, and $n_1 = 141$ to the Basal-like subtype. The dataset includes 753 microRNA (miRNA) and 5,784 gene expressions (Gene). Our screening algorithm identified 42 miRNA and 582 gene pairs, displayed in the upper left corner of Figure S1. Within the identified dense block, the strength of the association between miRNA and gene expression varies depending on the subtypes. Specifically, the association patterns in Luminal A & B are stronger than those in the Basal-like subtype.

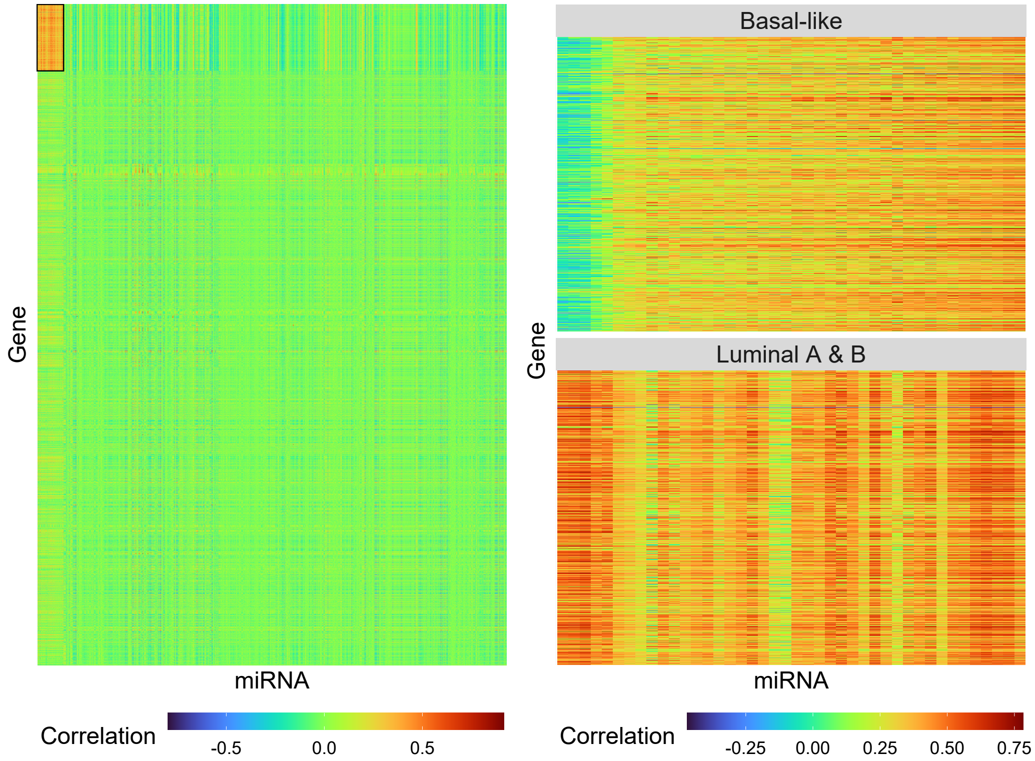

Fig. S1: Left: Heat map of the correlation matrix from all pairs of miRNA and gene expression. Right: Differential association pattern between miRNA and gene across subtypes. Specifically, Luminal A & B exhibit a stronger association than those of Basal-like.

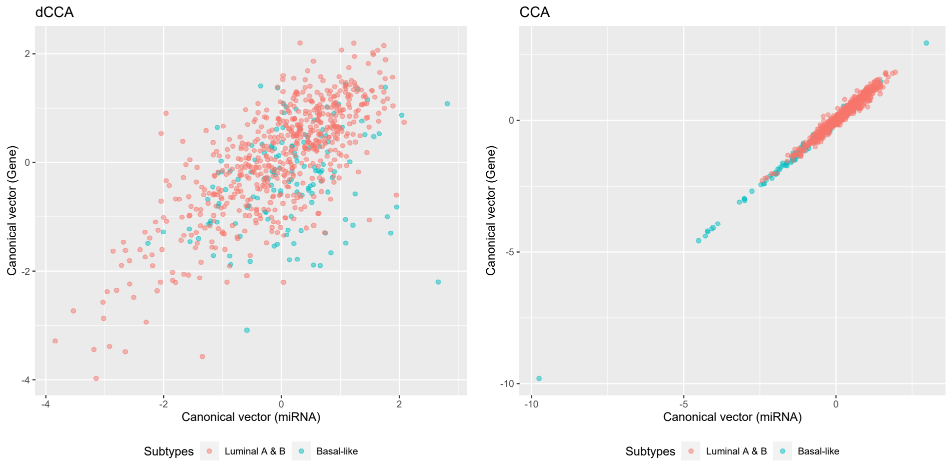

Fig. S2: Scatter plots of the canonical vectors (dCCA (left), and CCA (right), respectively).

The scatter plots of the canonical vectors are displayed in Figure S2. As shown in the heatmap, the association between miRNAs and genes is stronger in the Luminal subtypes (Luminal A '& B). The canonical vectors from dCCA reflect this differential pattern, where the slope of Luminal A & B is larger than that of Basal-like. However, CCA produces nearly identical canonical vectors. The difference in canonical correlation between the Luminals and Basal-like is significant: 0.4337 for dCCA (Luminal A & B: 0.7563, Basal-like: 0.3226) and -0.0083 for CCA (Luminal A & B: 0.9800, Basal-like: 0.9883). This demonstrates that dCCA can more effectively capture the differential association patterns between these clinical groups.

Validation of dCCA Result (TCGA-Pan Kidney)

In this section, we present the validation results of our findings in comparison with existing databases. Search results from two databases, `miRCancer` (Xie et al., 2013, `http://mircancer.ecu.edu`) and `dbDEMC` (Xu et al., 2022, `https://www.biosino.org/dbDEMC/index`), are shown in Table S2. Additionally, the results of the pathway analysis are detailed in Table S3.

Table S2. Database search results: miRCancer and dbDEMC are used to determine whether our selected miRNAs and genes have been reported in previous literature related to our subtypes.

Block	miRNA	Gene	RCC	RCCC	chRCC	ccRCC
1	mir126	NDUFA4L2	1	0	0	1
	mir145	FAM26E	1	0	0	1
	mir122	C3orf70	1	1	0	1
	mir143	KCNE4	1	0	1	1
	mir195	EDNRA	0	1	1	1
	mir338	EBF1	0	0	0	0
	mir497	STC2	1	0	1	1
	mir224	CDH13	1	1	0	1
	mir561	PDGFRB	0	0	0	0
	mir628	CDCA2	0	0	0	0
2	mir891	GALNT14	0	0	0	0
	mir892	USH1C	0	0	0	0
	mir891b	TMEM176A	0	0	0	0
	mir888	ACMSD	0	0	0	0
	mir874	SLC16A4	0	0	0	1
	mir141	GGT1	1	1	0	1
	mir892b	TMEM176B	0	0	0	0
	mir222	SLC28A1	0	0	1	0
	mir455	PNMA2	0	0	0	1
	mir221	TMEM200A	1	0	1	0
3	mir150	RAP1GA	0	0	0	0
	mir146b	C1R	1	0	0	0
	mir223	C1S	0	0	0	1
	mir10	NNMT	0	0	0	0
	mir21	CD2	1	0	0	1
	mir146	SIT1	0	0	0	0
	mir193	CD3E	0	0	0	0
	mir130	CD3D	0	0	0	0
	let7i	C3	0	0	0	0
	mir28	VSIG4	0	0	0	1

Table S3. Pathway analysis results. Note:**Reactome Immunoregulatory in the first column is Reactome Immunoregulatory interactions between a Lymphoid and a non-Lymphoid cell.**

	pvalue	qvalue	DS	DnS	NDS	NDnS	OR	logOR	Size	match_gene
GO:BP T cell activation	7.2E-05	0.135	4	26	2	568	43.69	3.7772	6	CD2/SIT1/CD3E/CD3D
GO:BP leukocyte activation	0.00016	0.135	4	26	3	567	29.08	3.3699	7	CD2/SIT1/CD3E/CD3D
GO:BP lymphocyte activation	0.00016	0.135	4	26	3	567	29.08	3.3699	7	CD2/SIT1/CD3E/CD3D
GO:BP cell activation	0.00031	0.195	4	26	4	566	21.77	3.0805	8	CD2/SIT1/CD3E/CD3D
KEGG Systemic lupus erythematosus	0.00534	1	3	27	5	565	12.56	2.5302	8	C1R/C1S/C3
**Reactome Immunoregulatory**	0.00534	1	3	27	5	565	12.56	2.5302	8	CD3E/CD3D/C3
Oncogenic BRCA1_DN.V1_UP	0.00534	1	3	27	5	565	12.56	2.5302	8	CDH13/SLC16A4/CD3D
Oncogenic ATF2_UP.V1_DN	0.00609	1	4	26	12	558	7.15	1.9677	16	NDUFA4L2/EDNRA/CDH13/PNMA2
GO:MF protein heterodimerization activity	0.00704	1	2	28	1	569	40.64	3.7048	3	CD3E/CD3D
Reactome Downstream TCR signaling	0.00704	1	2	28	1	569	40.64	3.7048	3	CD3E/CD3D
Reactome Phosphorylation of CD3 and TCR zeta chains	0.00704	1	2	28	1	569	40.64	3.7048	3	CD3E/CD3D
Reactome Translocation of ZAP-70 to Immunological synapse	0.00704	1	2	28	1	569	40.64	3.7048	3	CD3E/CD3D
KEGG Hematopoietic cell lineage	0.00774	1	3	27	6	564	10.44	2.3461	9	CD2/CD3E/CD3D
GO:BP regulation of T cell activation	0.0136	1	2	28	2	568	20.29	3.0099	4	SIT1/CD3E
Reactome TCR signaling	0.0136	1	2	28	2	568	20.29	3.0099	4	CD3E/CD3D
Reactome Generation of second messenger molecules	0.0136	1	2	28	2	568	20.29	3.0099	4	CD3E/CD3D
Reactome PD-1 signaling	0.0136	1	2	28	2	568	20.29	3.0099	4	CD3E/CD3D
Reactome Costimulation by the CD28 family	0.0136	1	2	28	2	568	20.29	3.0099	4	CD3E/CD3D
GO:BP cell surface receptor signaling pathway	0.0137	1	6	24	36	534	3.71	1.3106	42	EDNRA/STC2/CDH13/CD2/CD3E/C3
Oncogenic E2F1_UP.V1_DN	0.0183	1	3	27	9	561	6.93	1.9353	12	EBF1/CD3E/C3
GO:BP regulation of lymphocyte activation	0.022	1	2	28	3	567	13.5	2.6027	5	SIT1/CD3E
GO:MF serine-type endopeptidase activity	0.022	1	2	28	3	567	13.5	2.6027	5	C1R/C1S
GO:CC cell projection	0.022	1	2	28	3	567	13.5	2.6027	5	CDH13/USH1C
KEGG Primary immunodeficiency	0.022	1	2	28	3	567	13.5	2.6027	5	CD3E/CD3D
Reactome Initial triggering of complement	0.022	1	2	28	3	567	13.5	2.6027	5	C1S/C3
KEGG Complement and coagulation cascades	0.023	1	3	27	10	560	6.22	1.8281	13	C1R/C1S/C3
Oncogenic BMI1_DN_MEL18_DN.V1_DN	0.0283	1	3	27	11	559	5.65	1.731	14	TMEM176A/SLC16A4/TMEM176B
GO:BP membrane organization and biogenesis	0.032	1	2	28	4	566	10.11	2.3132	6	CDH13/CD2
GO:MF serine-type peptidase activity	0.032	1	2	28	4	566	10.11	2.3132	6	C1R/C1S
GO:MF serine hydrolase activity	0.032	1	2	28	4	566	10.11	2.3132	6	C1R/C1S
Reactome Complement cascade	0.032	1	2	28	4	566	10.11	2.3132	6	C1S/C3
Oncogenic MEL18_DN.V1_DN	0.0342	1	3	27	12	558	5.17	1.6422	15	TMEM176A/SLC16A4/TMEM176B
GO:BP immune system process	0.0352	1	4	26	22	548	3.83	1.3434	26	CD2/SIT1/CD3E/CD3D
GO:BP signal transduction	0.0372	1	8	22	73	497	2.48	0.9065	81	EDNRA/STC2/CDH13/PDGFRB/CD2/SIT1/CD3E/C3
GO:BP regulation of immune system process	0.0434	1	2	28	5	565	8.07	2.0883	7	SIT1/CD3E
GO:MF endopeptidase activity	0.0434	1	2	28	5	565	8.07	2.0883	7	C1R/C1S
GO:MF protein dimerization activity	0.0434	1	2	28	5	565	8.07	2.0883	7	CD3E/CD3D
KEGG T cell receptor signaling pathway	0.0434	1	2	28	5	565	8.07	2.0883	7	CD3E/CD3D
Oncogenic BMI1_DN.V1_DN	0.0434	1	2	28	5	565	8.07	2.0883	7	TMEM176A/TMEM176B

References

Tsourakakis, C., Bonchi, F., Gionis, A., Gullo, F., and Tsiarli, M. (2013). Denser than the densest subgraph: extracting optimal quasi-cliques with quality guarantees. In *Proceedings of the 19th ACM SIGKDD International Conference on Knowledge Discovery and Data Mining*, KDD '13, page 104–112, New York, NY, USA. Association for Computing Machinery.

Wu, Q., Huang, X., Culbreth, A. J., Waltz, J. A., Hong, L. E., and Chen, S. (2022). Extracting brain disease-related connectome subgraphs by adaptive dense subgraph discovery. *Biometrics*, 78(4):1566–1578.

Xie, B., Ding, Q., Han, H., and Wu, D. (2013). miRCancer: a microRNA–cancer association database constructed by text mining on literature. *Bioinformatics*, 29(5):638–644.

Xu, F., Wang, Y., Ling, Y., Zhou, C., Wang, H., Teschendorff, A. E., Zhao, Y., Zhao, H., He, Y., Zhang, G., and Yang, Z. (2022). dbdemc 3.0: Functional exploration of differentially expressed mirnas in cancers of human and model organisms. *Genomics, Proteomics & Bioinformatics*, 20(3):446–454. Bioinformatics Commons—2022.
